# Double balloon enteroscopy in the detection and removal of jejunal anisakiasis: Case report

**DOI:** 10.1002/deo2.339

**Published:** 2024-02-13

**Authors:** Erika Abe, Tsukasa Ishida, Tatsuya Osuga, Saori Kakuyama, Hirofumi Ogawa, Katsutoshi Nabeshima

**Affiliations:** ^1^ Department of Gastroenterology Akashi Medical Center Hyogo Japan; ^2^ Department of Gastroenterology Takatsuki General Hospital Osaka Japan

**Keywords:** acute abdomen, double‐balloon enteroscopy, jejunal anisakiasis, raw fish consumption, small bowel obstruction

## Abstract

A 53‐year‐old Japanese man presented to the emergency department with epigastric pain and bloating. Computed tomography revealed a thickening of the jejunal wall and proximal dilation. Double‐balloon enteroscopy was performed to investigate the jejunal thickening, which revealed an anisakis larva in the jejunum with an associated ulcer. The larva was removed using endoscopic forceps, after which there was immediate improvement of symptoms. Further patient interview determined that he had consumed marinated mackerel the day before the onset of symptoms. After diagnosis of small intestinal anisakiasis, he was successfully treated using double‐balloon enteroscopy. Its use for small intestinal anisakiasis is rare, and this case may be the first instance in the jejunum. Removal of the anisakis larva led to a clear diagnosis and a quick resolution of symptoms. A history of raw fish consumption a few days before the onset of abdominal symptoms and abnormal findings on computed tomography scans are key to the diagnosis of small intestinal anisakiasis. Double‐balloon enteroscopy was thought to be a safe means of making accurate diagnoses and appropriate treatment of our patients.

## INTRODUCTION

Anisakiasis is a zoonotic parasitic disease caused by the consumption of raw or undercooked seafood infected with Anisakis third‐stage larvae (L3). If humans consume infected raw or undercooked fish, they can become incidental hosts. The L3 larvae enter the digestive duct and cause anisakiasis.^1^

Japan has the highest number of reported cases, with approximately 2000–3000 cases annually, in part because of the commonality of uncooked seafood.^1^ Sushi and *sashimi*, commonly consumed in Japan, are often the source of human infection. As these food cultures spread globally and delivery systems expand overseas, anisakiasis could become a worldwide problem.

Most cases of anisakiasis are found in the stomach, and intestinal anisakiasis is rare. One reason for its obscurity could be the difficulty in approaching and diagnosing it. Many cases of intestinal anisakiasis have likely been surgically resected without appropriate diagnosis. Intestinal anisakiasis carries a risk of perforation, so early diagnosis is considered to be important. The endoscopic approach to the small intestine has become more accessible with the introduction of capsule and balloon‐assisted enteroscopy.^2^ Especially double‐balloon enteroscopy (DBE) could be used in diagnosis and therapy. We report a case in which jejunal anisakiasis was successfully diagnosed and treated by double‐balloon enteroscopy.

## CASE REPORT

**FIGURE 1:**
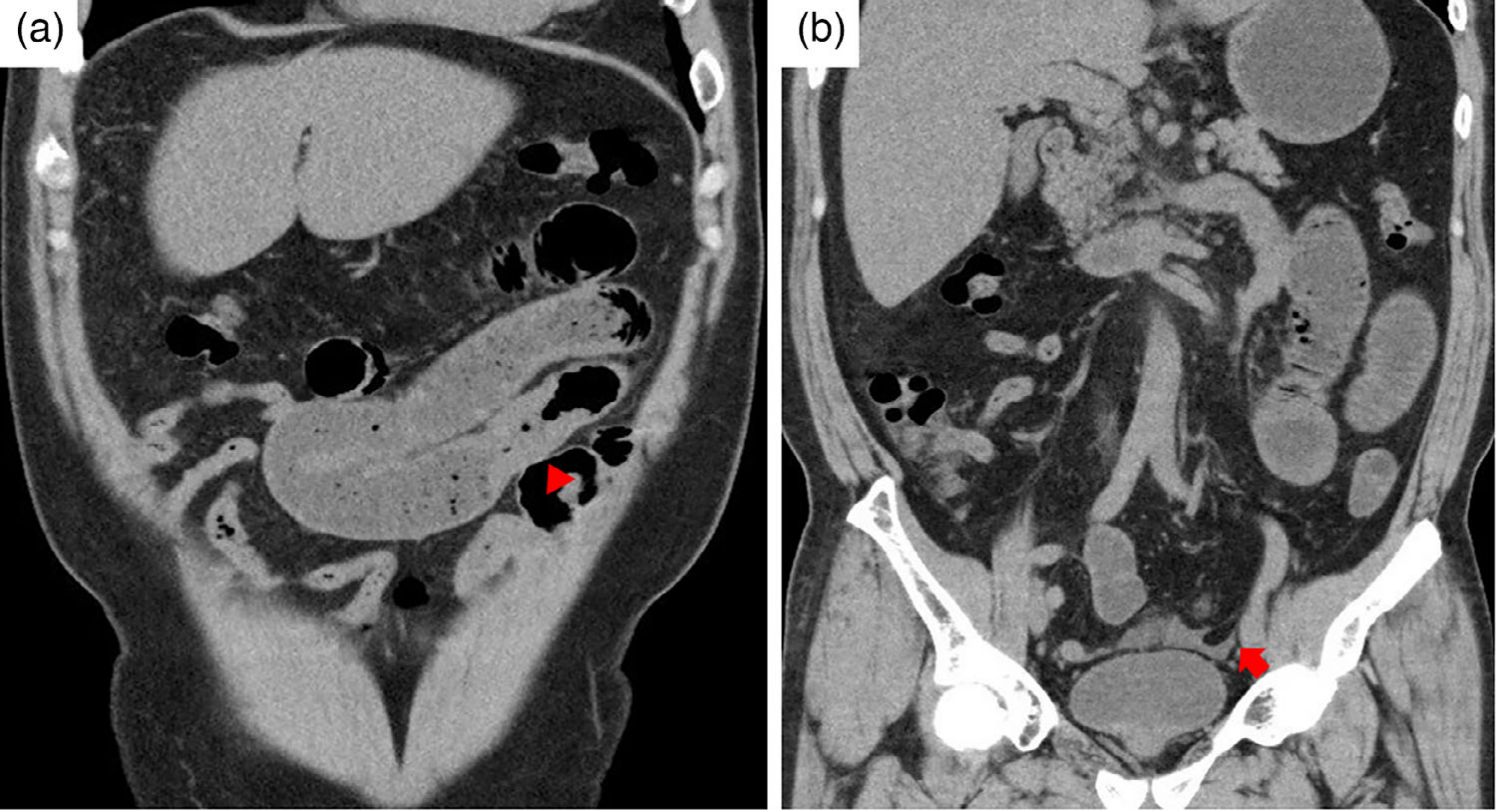
Plain‐computed tomography scan on the day of admission showed edematous wall thickening of the jejunum (red triangle), dilation of the proximal side (a), and a small amount of ascites (red arrow) (b).

**FIGURE 2:**
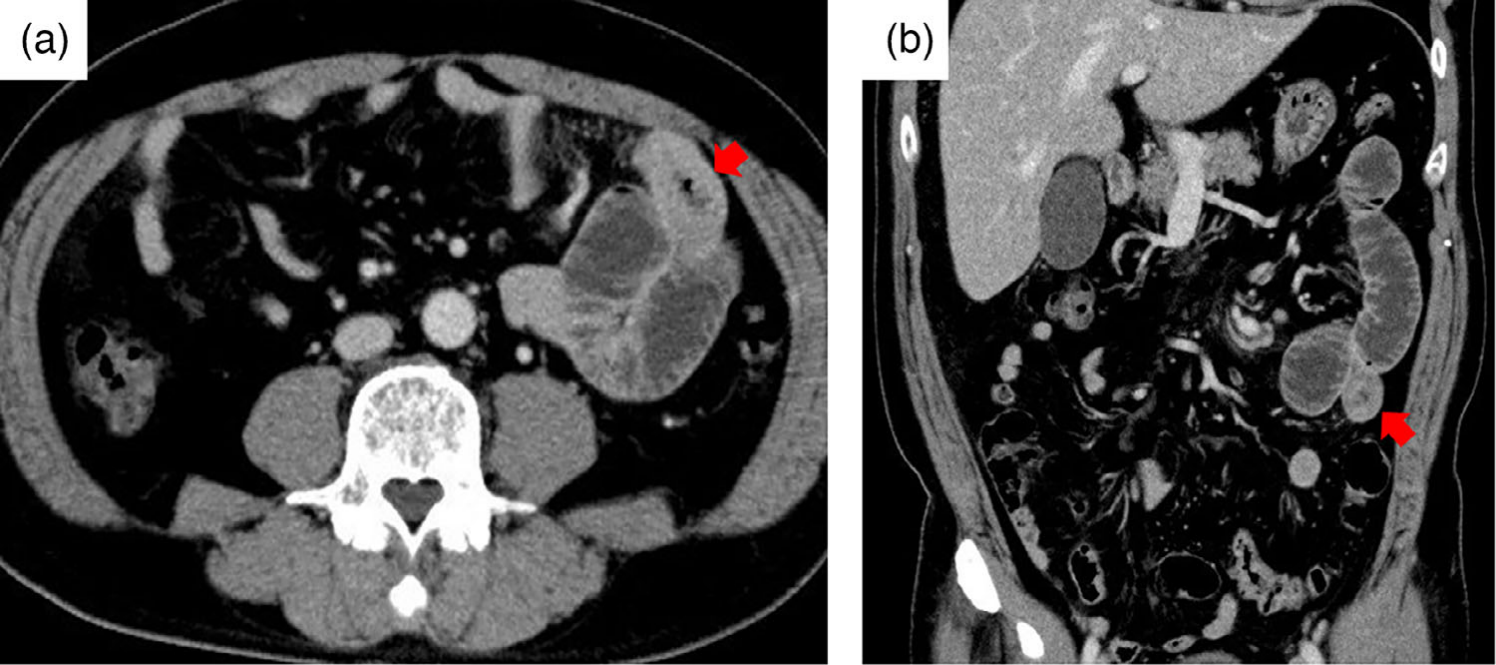
Contrast‐enhanced computed tomography on the second day of admission showed circumferential wall thickening with contrast effect in the partial segment of the small intestine (red arrow) (a, b).

A 53‐year‐old Japanese man presented to our emergency department with epigastric pain and bloating continuing for two days. Laboratory tests showed a high white blood cell count (9300 cells/mL) with a normal eosinophil count (2.8%) and C‐reactive protein (8.84 mg/dL). Small bowel gas was observed on abdominal radiography, and abdominal computed tomography (CT) showed approximately 5 cm edematous wall thickening of the jejunum with ascites and dilation of the proximal side (Figure 1). He was admitted for investigation of the jejunal thickening. On admission, he had a bowel obstruction and difficulty in defecation. Despite no history of abdominal surgery, he had been regularly using a COX‐2 inhibitor for cervical spondylotic radiculopathy. Potential causes of the obstruction were evaluated, including the possibility of non‐steroidal anti‐inflammatory drug‐induced enteropathy or other underlying conditions. Contrast‐enhanced CT showed circumferential wall thickening with contrast effect in a partial segment of the small intestine (Figure 2a,b).

**FIGURE 3:**
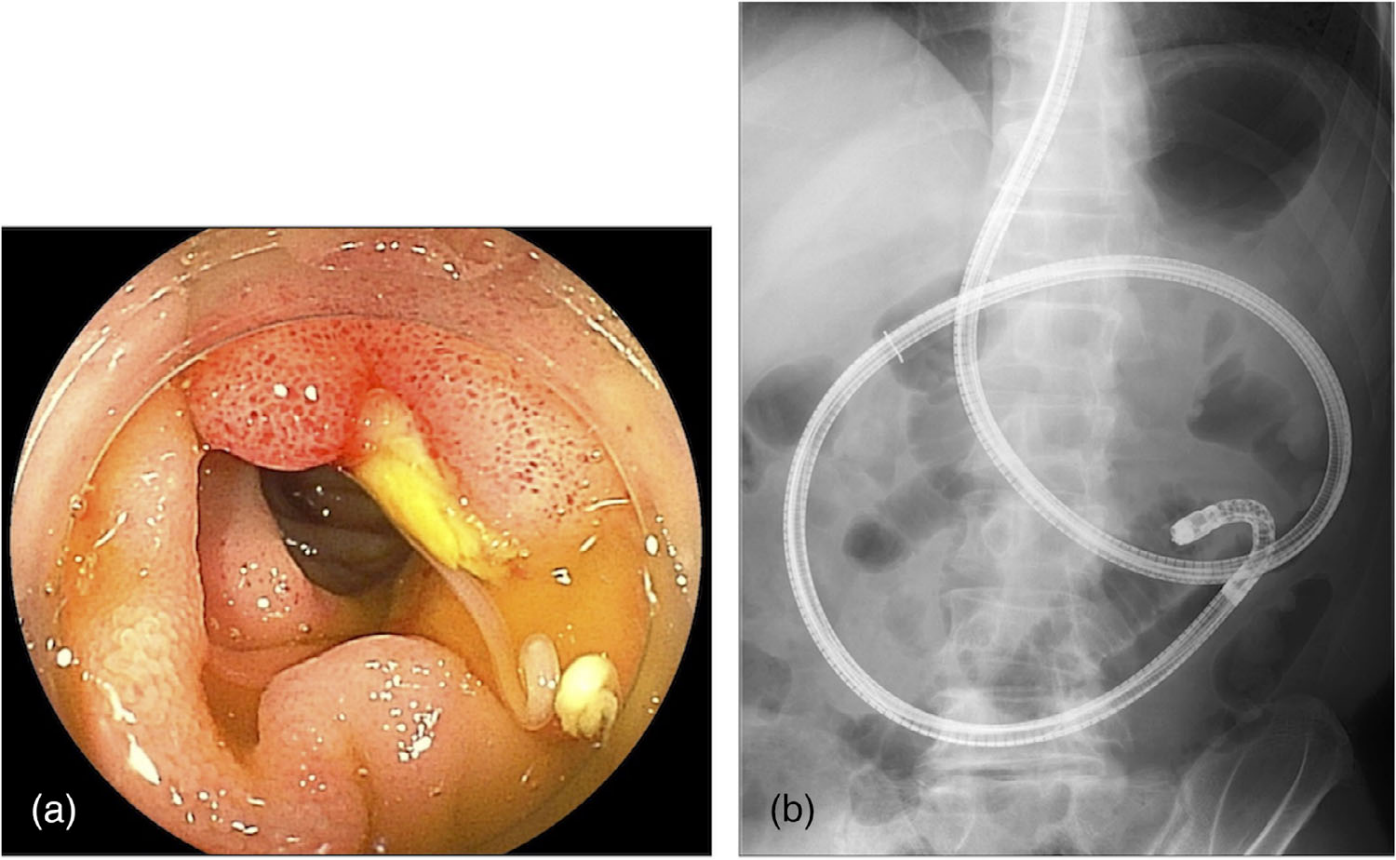
Double‐balloon small bowel endoscopy on the fourth day of admission revealed an anisakis larva in an ulcer (a) and scope position of fluoroscopy (b).

**FIGURE 4:**
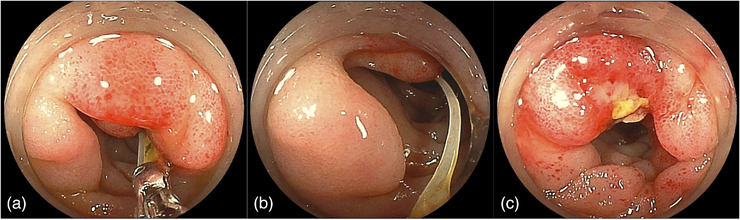
Anisakis larva was successfully removed by enteroscopy using endoscopic forceps (a–c).

The patient fasted until the fourth day of hospitalization, then underwent DBE (EI‐580BT; Fujifilm) by antegrade approach. DBE revealed an ulcerative lesion with surrounding edematous mucosa in the jejunum. An anisakis larva was found at the center of the ulcer (Figure 3a,b), which was subsequently removed using endoscopic forceps (Figure 4a–c). No other abnormal lesions were found within the range of observation or according to CT, so we did not examine the distal intestine beyond the area where the anisakis larvae had been detected. Fortunately, the symptoms resolved quickly after the removal of the anisakis larva and he was discharged without any worsening of symptoms after eating on the eighth day of hospitalization. After discharge, no further symptoms were observed. He opted against further CT scans or serum immunological tests to exclude anisakis larvae after becoming asymptomatic. In further interviews, he disclosed for the first time that he had consumed marinated mackerel one day before the onset of symptoms, and this was thought to be the source of the issue. He was featured in this case report after we received informed consent to the inclusion of the details of his case.

## DISCUSSION

Anisakiasis is a human parasitic disease caused by third‐stage anisakis larvae found in fish and squids as intermediary hosts. While most cases of anisakiasis have been reported as gastric anisakiasis, many patients with intestinal anisakiasis are likely misdiagnosed with other diseases, such as wall thickening, peritonitis, perforation, bleeding, or intussusception. Among the 201 cases of reported bowel anisakiasis in Japan that were identified using the Japanese Diagnosis Procedure Combination in‐patient database, 50.7% had bowel obstruction, 8.0% had perforation or peritonitis, 2.0% had intestinal bleeding, and one case had intussusception.^3^ Misdiagnoses and lack of awareness have likely contributed to the relatively low number of reported cases of intestinal anisakiasis. In a single‐center retrospective study, when the diagnostic criteria for intestinal anisakiasis included a recent history of raw fish consumption, anisakis antibody, and CT scan (gastric anisakiasis required endoscopic diagnosis), 53% were diagnosed with small intestinal anisakiasis (SIA) and 47% with gastric anisakiasis.^4^ SIA may therefore be more common than previously thought.

The symptoms of SIA include abdominal pain, vomiting, nausea, and/or diarrhea. There are two main mechanisms involved: allergic reaction and direct tissue damage due to penetration of the anisakis larvae.^1^ The abdominal symptoms therefore tend to develop more slowly in cases of intestinal anisakiasis compared with gastric cases. Actually, in gastric anisakiasis, symptoms peak within 6 h after consuming raw fish, while in SIA, they peak within 48 h.^4^ Obtaining the clinical history from the past few days is therefore vital for diagnosing intestinal anisakiasis. Additionally, imaging modalities such as CT and ultrasonography are useful in aiding the diagnosis. Gastric anisakiasis can be diagnosed by endoscopically observing the larvae. Another method is the immunological test, such as anisakis‐specific immunoglobulin A (IgA), IgG, and IgE, but it is not very useful due to the time it takes to obtain results. In this case, we did not check the serum immunological test because there was no suspicion of SIA because the patient did not originally mention his recent consumption of raw fish. Although DBE and capsule endoscopy can be used in the diagnosis of SIA, endoscopic diagnosis of SIA remains more challenging than gastric anisakiasis, mainly due to the greater distance from the oral or anal route. Consequently, they have only been employed in a limited number of cases.^5^, ^6^, ^7^

Treatment for gastric anisakiasis involves the removal of the anisakis larvae. However, in the case of SIA, there is not yet an established standard therapy. Among 201 cases of bowel anisakiasis in the Japanese database, 14 cases (7.0%) underwent open surgery, three (1.5%) underwent colonoscopic removal of Anisakis larvae, and others could be managed with conservative treatments.^3^ In almost all cases of intestinal anisakiasis, conservative treatments are successful because anisakis larvae typically survive only for a few days in the intestinal tract, resulting in inflammation subsiding within 2–3 weeks.^8^ Nevertheless, severe stenosis caused by inflammation in SIA may require surgical treatment.^9^ If the intestinal wall is irreversibly damaged by the inflammation of anisakis larvae, surgery may be performed after about 3 weeks during the preservation period, leading to a prolonged hospital stay. On the other hand, treatment involving the removal of anisakis larvae by DBE can result in a quick resolution of symptoms.^6^, ^7^ The use of DBE for SIA has been reported in two Japanese cases.^6^, ^7^ In English literature, anisakiasis of the ileum removed by colonoscopy has been reported.^10^ Although the use of DBE is relatively rare, it means surgery can be avoided and there will be a shorter treatment period. The overall complications associated with DBE, including perforation, bleeding, and pancreatitis, have been reported to be around 1%.^2^ In contrast, intestinal anisakiasis has a higher risk of perforation or peritonitis (8%) and bleeding (2%).^3^ There have been no reported complications from DBE for SIA.^6^, ^7^ However, CT is required to determine the absence of perforation and the location of the abnormal lesion before intervention. We suggest that DBE should be performed in only experienced and sufficiently equipped facilities.

In conclusion, our patient with SIA was successfully diagnosed and treated using DBE. The use of DBE for SIA is rare, and this case may be the first such instance where DBE was used for the treatment of a patient with SIA in the jejunum. Correct diagnosis and the removal of the anisakis larva can lead to a quick resolution of symptoms. A history of raw fish consumption a few days before the onset of abdominal symptoms and abnormal bowel findings on CT scans are key to the diagnosis of small bowel anisakiasis. DBE was a useful tool in both the diagnosis and treatment of SIA.

## CONFLICT OF INTEREST STATEMENT

None.

## ETHICS STATEMENT

This case report was conducted in accordance with the ethical standards established in the 1964 Declaration of Helsinki and its later amendments.
